# Cell–Biomaterial Interactions

**DOI:** 10.3390/bioengineering10020241

**Published:** 2023-02-11

**Authors:** Vincent Deplaigne, Gael Y. Rochefort

**Affiliations:** SATT Lutech TTO, 75001 Paris, France

In animals, the extracellular matrix (ECM) forms a three-dimensional network occupying the intercellular spaces (interstitial matrix) or serving as physical and biochemical support for cells and tissues (basement membrane). Presenting a highly variable nature and composition, according to the cells having produced it and according to the tissues, the ECM is broadly composed of an organic fraction represented by the extracellular macromolecules (collages, proteoglycans, elastin, structural glycoproteins or more enzymes) and a mineral fraction [1]. The intercellular interstitial spaces are thus essentially filled by gels of polysaccharides and fibrous proteins forming a loose three-dimensional network acting as a mechanical shock absorber against the compressive stresses exerted on the ECM. The ECM of basal membranes consists of an apposition of several sheets upon which are various connective tissue and epithelial cells, and it presents a specific composition from one tissue to another: essentially, collagen and hydroxyapatite in the bone tissue; reticular, elastic and collagenous fibers in loose connective tissues [2]; and globular proteins suspended in blood plasma. The biochemical nature of the natural and native extracellular matrix, its composition, its organization, its heterogeneity or even its roughness are all elements providing microstructural, mechanical and biochemical signals that can influence cell adhesion and behavior, inter-cellular communication or cell differentiation, and this is achieved in a static or dynamic way, as well as spatially or temporally [3].

The ability of different Implantable exogenous (bio)materials, synthetic or not, to mimic the complex interactions between cells and their microenvironment in vivo is essential to the successful implantation of a biomaterial [4]. These interactions, between the biomaterial and the cells, are conditioned by their reciprocal orientation, the three-dimensional architecture, the zone and the type of contact at the microscopic scale at the cell/(bio)material interface, and above all, the ability of this contact to induce an appropriate cellular response. Understanding and mastering these different constraints in the design of biomaterials to be implanted are therefore essential for the development of the next generation of tissue engineering [5].

In the context of advanced approaches in this field, the current Special Issue of *Bioengineering* aimed to gather modern studies related to cell–biomaterial interactions. The current Special Issue included two original articles and five review articles.

Among the different fibrous proteins structuring the ECM of connective tissues, elastin and collagen represent the most abundant proteins of the ECM of adipose tissue [6]. Newman and her colleagues thus studied the effect of different scaffolds made up of collagen and elastin, as well as their physico-chemical and mechanical properties, on the induction of adipogenic differentiation of stem cells derived from human adipose tissue [7]. A study by FTIR spectrometry also showed the existence of secondary binding interactions between collagen and elastin, while a porous structure was visualized by electron microscopy within all the scaffolds. The authors then reported that the increase in the final concentrations of collagen and elastin, associated with the presence of cross-linking, made it possible to reduce the rate of water swelling of the scalds while improving their modulus of elasticity and their resistance to compression. Specific cell morphological behaviors were subsequently reported depending on the type of scaffold used, since smeared morphologies were visualized when using softer, non-cross-linked scaffolds, while cells exhibited spheroid morphology and better induction of adipogenic differentiation when stiffer and/or cross-linked elastin–collagen-based scaffolds were used. In conclusion, this study highlighted the importance of the mechanical properties of a collagen and elastin scaffold in the induction of a specific cell morphology and a better induction of adipogenic differentiation, allowing researchers to create more physiologically relevant three-dimensional in vitro culture models [8,9].

An alteration in the biology and mechanical properties of the ECM has already been demonstrated during the process of tumor progression, inducing a modification of the proliferation of tumor cells and a modulation of their gene expression associated with a modification of the actin cytoskeleton [10]. In the second article, Sugimoto and his colleagues demonstrated a new target gene coding for a Yes-associated protein (YAP) [11,12], which can be modulated by matrix metalloproteinase (MMP)-24 and is involved in tumor cell mechanotransduction in response to a modification of the MEC [13]. The authors first showed an increase in MMP24 expression in MCF-7 human breast cancer cells when these cells were cultured on stiffer substrates, while MMP24 expression was significantly reduced by knockdown of YAP. The authors thus concluded that MMP24 could negatively regulate the aggressiveness of cancer cells in the rigid ECM environment during tumor progression, and the authors were thus able to show that the stiffening of an ECM favored the invasion of tumor cells [14].

In the next article, Chang and Lin reviewed the various hydrogel-based in vitro tumor models and methods for generating gradient stiffness to study migration and other fate processes of cancer cells in the pancreatic ductal adenocarcinoma [15]. This pathology, particularly aggressive and resistant to chemotherapy, due to the presence of dense fibrous tissue, hypovascularized and composed of stromal cells and extracellular matrices, is the most common type of pancreatic cancer and has known only modest improvements in patient survival rates over the past few decades. Other studies have concluded that increased ECM stiffness also triggers the invasion of tumor cells in the pancreatic ductal adenocarcinoma [16,17].

Tysan and colleagues then reviewed the mechanisms conditioning the transition from a smooth muscle vascular phenotype to an osteogenic phenotype during vascular calcification [18]; characterized by the hardening of the arteries, vascular calcification is the deposition of hydroxyapatite crystals in arterial tissue. Among the different initiation pathways and mechanisms behind vascular calcification, the authors specify in particular the involvement of the wingless-related integration site (WNT) signaling pathway, as well as bone morphogenic proteins (BMPs) and mechanical stress. The authors rightly postulated that developing a better understanding of the mechanisms behind calcification could lead to the development of a potential treatment in the future [18].

Fuest and his colleagues then examined the different perspectives and challenges surrounding the bioprinting technique in the specific case of the cornea [19]. Indeed, currently, corneal transplantation remains the ultimate treatment option for advanced stromal and endothelial disorders.

Always focused on the eye and its different tissues, Baino and Kargozar then address the different aspects of the regulation and modulation of the response of ocular cells/tissues in the context of the use of implantable biomaterials and drug delivery systems [20]. Indeed, ocular drug delivery systems, allowing sustained release while maintaining therapeutic drug levels in the target tissues, must allow the use of an encapsulated drug while delivering the appropriate concentration of the drug to the target tissue. Their review article provides an overview of biomaterials used as drug carriers in the eye, including micro- and nanospheres, liposomes, hydrogels, and multi-implant materials. Furthermore, the advantages and limitations of these devices are discussed with reference to the main ocular applications [20].

Finally, Zhao and colleagues reviewed the various aspects of hepatic stem cell differentiation in 2D and 3D biomaterial systems [21]. Indeed, there is a critical shortage of donor livers for the treatment of end-stage liver failure implying the urgent need for alternative treatment options. Hepatocyte-like cells derived from various stem cells represent a promising cell source for hepatocyte transplantation, liver tissue engineering, and bioartificial liver assist device development. To further promote liver differentiation and maturation, biomaterials can be designed to recapitulate cell–extracellular matrix interactions in both 2D and 3D configurations. In this latest review, the authors summarize and compare the various 2D and 3D biomaterial systems that have been applied to liver differentiation, highlighting their roles in presenting biochemical and physical cues to different stem cell sources.

Contributions to this Special Issue take readers on a journey into topical research activities in the specific area of interactions between cells and biomaterials, covering new and different aspects, within different tissues and cells, as well as during tumor progression processes. As guest editor for this Special Issue, I am optimistic on the fact that this specific area of research will again spark inspiration and ideas for further research and development in the field. In this way, more data will be collected, highlighting significant aspects that can be used in therapy, and at the same time improving the application of these advanced methods in terms of economy and quality.

## Data Availability

No sensitive data was included in the manuscript.

## References

[1] Kyriakopoulou K., Piperigkou Z., Tzaferi K., Karamanos N.K. (2022). Trends in extracellular matrix biology. Mol. Biol. Rep..

[2] Rochefort G.Y., Pallu S., Benhamou C.-L. (2010). Osteocyte: The unrecognized side of bone tissue. Osteoporos. Int..

[3] Fattahi R., Chamkhorami F.M., Taghipour N., Keshel S.H. (2022). The effect of extracellular matrix remodeling on material-based strategies for bone regeneration: Review article. Tissue Cell.

[4] Deplaigne V., Rochefort G.Y. (2021). Bone tissue engineering at a glance. AIMS Bioeng..

[5] Collignon A.-M., Lesieur J., Vacher C., Chaussain C., Rochefort G.Y. (2017). Strategies Developed to Induce, Direct, and Potentiate Bone Healing. Front. Physiol..

[6] Keck M., Haluza D., Selig H.F., Jahl M., Lumenta D.B., Kamolz L.-P., Frey M. (2011). Adipose tissue engineering: Three different approaches to seed preadipocytes on a collagen-elastin matrix. Ann. Plast. Surg..

[7] Newman K., Clark K., Gurumurthy B., Pal P., Janorkar A.V. (2020). Elastin-Collagen Based Hydrogels as Model Scaffolds to Induce Three-Dimensional Adipocyte Culture from Adipose Derived Stem Cells. Bioengineering.

[8] Alharbi Z., Almakadi S., Opländer C., Vogt M., Rennekampff H.-O., Pallua N. (2014). Intraoperative use of enriched collagen and elastin matrices with freshly isolated adipose-derived stem/stromal cells: A potential clinical approach for soft tissue reconstruction. BMC Surg..

[9] Sawadkar P., Mandakhbayar N., Patel K.D., Buitrago J.O., Kim T.H., Rajasekar P., Lali F., Kyriakidis C., Rahmani B., Mohanakrishnan J. (2021). Three dimensional porous scaffolds derived from collagen, elastin and fibrin proteins orchestrate adipose tissue regeneration. J. Tissue Eng..

[10] Fullár A., Dudás J., Oláh L., Hollósi P., Papp Z., Sobel G., Karászi K., Paku S., Baghy K., Kovalszky I. (2015). Remodeling of extracellular matrix by normal and tumor-associated fibroblasts promotes cervical cancer progression. BMC Cancer.

[11] Uchihara T., Miyake K., Yonemura A., Komohara Y., Itoyama R., Koiwa M., Yasuda T., Arima K., Harada K., Eto K. (2020). Extracellular Vesicles from Cancer-Associated Fibroblasts Containing Annexin A6 Induces FAK-YAP Activation by Stabilizing β1 Integrin, Enhancing Drug Resistance. Cancer Res..

[12] Wang W., Wu L., Tian J., Yan W., Qi C., Liu W., Xuan S., Shang A. (2022). Cervical Cancer Cells-Derived Extracellular Vesicles Containing microRNA-146a-5p Affect Actin Dynamics to Promote Cervical Cancer Metastasis by Activating the Hippo-YAP Signaling Pathway via WWC. J. Oncol..

[13] Sugimoto W., Itoh K., Hirata H., Abe Y., Torii T., Mitsui Y., Budirahardja Y., Tanaka N., Kawauchi K. (2020). MMP24 as a Target of YAP Is a Potential Prognostic Factor in Cancer Patients. Bioengineering.

[14] Giussani M., Merlino G., Cappelletti V., Tagliabue E., Daidone M.G. (2015). Tumor-extracellular matrix interactions: Identification of tools associated with breast cancer progression. Semin. Cancer Biol..

[15] Chang C.-Y., Lin C.-C. (2021). Hydrogel Models with Stiffness Gradients for Interrogating Pancreatic Cancer Cell Fate. Bioengineering.

[16] Ufuk A., Garner T., Stevens A., Latif A. (2022). Monocarboxylate Transporters Are Involved in Extracellular Matrix Remodelling in Pancreatic Ductal Adenocarcinoma. Cancers.

[17] Begum A., Ewachiw T., Jung C., Huang A., Norberg K.J., Marchionni L., McMillan R., Penchev V., Rajeshkumar N.V., Maitra A. (2017). The extracellular matrix and focal adhesion kinase signaling regulate cancer stem cell function in pancreatic ductal adenocarcinoma. PLoS ONE.

[18] Tyson J., Bundy K., Roach C., Douglas H., Ventura V., Segars M., Schwartz O., Simpson C. (2020). Mechanisms of the Osteogenic Switch of Smooth Muscle Cells in Vascular Calcification: WNT Signaling, BMPs, Mechanotransduction, and EndMT. Bioengineering.

[19] Fuest M., Yam G.H.-F., Mehta J.S., Campos D.F.D. (2020). Prospects and Challenges of Translational Corneal Bioprinting. Bioengineering.

[20] Baino F., Kargozar S. (2020). Regulation of the Ocular Cell/Tissue Response by Implantable Biomaterials and Drug Delivery Systems. Bioengineering.

[21] Zhao X., Zhu Y., Laslett A.L., Chan H.F. (2020). Hepatic Differentiation of Stem Cells in 2D and 3D Biomaterial Systems. Bioengineering.

